# Working with laboratory rodents in Spain: a survey on welfare and wellbeing

**DOI:** 10.1186/s42826-021-00098-w

**Published:** 2021-07-27

**Authors:** Olatz Goñi-Balentziaga, Iván Ortega-Saez, Sergi Vila, Garikoitz Azkona

**Affiliations:** 1 Department of Clinical and Health Psychology and Research Methodology, Euskal Herriko Unibertsitatea (UPV/EHU), Donostia, Spain; 2 Scientific and Technological Centers (CCIT), University of Barcelona (UB), Barcelona, Spain; 3 Department of Basic Psychological Processes and their Development, Euskal Herriko Unibertsitatea (UPV/EHU), Donostia, Spain

**Keywords:** Environmental enrichment, Analgesia, Anesthesia, Euthanasia, Human-animal interaction, Social support, Laboratory rodent

## Abstract

**Background:**

Replacement, reduction and refinement, the 3R principles, provide a framework to minimize the use and suffering of animals in science. In this context, we aimed to determine the actual perception that individuals working with laboratory rodents in biomedical research have on animal welfare and on their interaction with the animals, as well as how they perceive its impact on their social relations. To this end, we designed an anonymous on-line survey for people working with rodents, at three responsibility levels, in Spain.

**Results:**

Of the 356 participants, 239 were women (67 %); 263 were researchers (74 %), and 93 animal facility staff (26 %), of which 55 were caretakers/technicians (15 %), and 38 welfare officer/veterinarians (11 %). Animal facility staff indicated environmental enrichment to be a universal practice. About half of the participants reported that, in their opinion, animals suffer “little to none” or “minor” stress and pain. Animal caretakers/technicians and researchers perceived higher levels of stress and pain than welfare officers/veterinarians. Participants judged decapitation the most unpleasant method to kill rodents, whereas anaesthetic overdose was the least one. A sizable proportion − 21 % of animal caretakers/technicians and 11.4 % of researchers - stated that they were never given the choice not to euthanize the rodents they work with. Overall, women reported higher interactions with animals than men. Nevertheless, we could detect a significant correlation between time spent with the animals and interaction scores. Notably, 80 % of animal facility staff and 92 % of researchers rarely talked about their work with laboratory rodents with people outside their inner social circle.

**Conclusions:**

Overall, the participants showed high awareness and sensitivity to rodent wellbeing; animal facility staff reported a similar perception on welfare questions, independently of their category, while researchers, who spent less time with the animals, showed less awareness and manifested lower human-animal interaction and less social support. Regarding the perception on social acceptance of laboratory animal work, all groups were cautious and rarely talked about their job, suggesting that it is considered a sensitive issue in Spain.

**Supplementary Information:**

The online version contains supplementary material available at 10.1186/s42826-021-00098-w.

## Background

The Animal Welfare Committee established *the five freedoms* as general indicators of animal welfare. These freedoms refer to the fact that all animals must be kept free from hunger and thirst, from discomfort, from pain, injury or disease, free to express the natural behaviors of the species, and from fear or suffering [1]. Unfortunately, scientific research directly affects each of them. That is why one important challenge of biomedical research is to try to reduce the tension between the potential benefit of scientific results and the welfare of the animals used. European (Directive 2010/63/UE) and Spanish legislations (RD53/2013) are based on replacement, reduction and refinement (3Rs). These principles state that if animals have to be used in experiments, researchers should made every effort to replace them with non-sentient alternatives, to reduce them to a minimum, and to refine experiments and housing conditions causing the minimum pain or distress [2].

Research animals can experience distress due to poor husbandry, handling techniques, ineffective euthanasia methods, or as an unavoidable consequence of the procedure employed, for example, unrelieved pain in an experiment designed to test the effectiveness of a pain-killing drug [2, 3]. Efforts to refine and optimize the care and use of animals in research have been ongoing for many years and have led to general standardization of rodent models, particularly with regard to animal housing, genetics, and health status. Concurrently, different recommendations have been published with the intent of promoting general animal wellbeing through the enrichment of their home cage environment [4–7]. In the same way, guides to laboratory rodent analgesia and anesthesia are readily available [8]. Although it has been reported that current scientific literature does not provide sufficient details on this regard [9], an increase in the reported administration of analgesia and anesthesia to laboratory rodents undergoing surgical procedures was observed in the past decade [10, 11]. In line with this, our previous work showed that researchers working with laboratory rodents in Spain are fully aware of the pain that a surgical procedure involves and that the use of analgesia and inhalation anesthesia is a common practice in Spain [12].

The vast majority of people who work with laboratory rodents must sometimes euthanize them for scientific or ethical reasons. The optimal choice of euthanasia method depends on a number of factors, including the scientific goals of the study, the need to minimize animal pain and/or distress, applicable guidelines and laws, the training and proficiency of personnel, and the safety and emotional needs of the personnel performing the euthanasia [13]. Killing an animal is physicologically stressful to the person who performs it [14] and people can develop euthanasia stress, a concept of being aware and psychologically challenged when faced with the task of euthanizing animals [15]. In Spain, two studies have explored the unpleasantness of the killing methods for rodents described in the Spanish legislation, and participants in both surveys reported that drug overdose is the least unpleasant method [12, 16].

 Currently, in many institutions a team of animal caretakers, technicians, welfare officers and veterinarians (animal facility staff) provide the husbandry and care of the laboratory animals. Therefore, it is not unusual that caretakers and technicians assume responsibility for specific groups of animals, overseeing them for long periods of time and establishing relationships with them. Researchers, on the other hand, often interact with animals only when conducting a specific procedure [17]. Human-animal interaction is important in animal experimentation both for the welfare of the animal and for the outcome of the experiment, since it has been shown that, for example, gentle handling facilitates behavioral testing and good data collection, and improves animal welfare [18, 19]. In the same way, the interaction with laboratory animals has an overwhelming impact on the emotional health of the staff. This emotional impact is exacerbated by the responsibilities of working with other sentient beings and determining how best to ensure their wellbeing, particularly following interventions that cause a certain degree of harm or distress [20]. A recent study showed that the professional quality of life of laboratory animal personnel is associated with animal stress/pain, enrichment diversity/frequency, euthanasia method and control, and social support [21].

Public opinion on the use of laboratory animals depends on a myriad of biological and sociocultural factors, ranging from people’s gender and age to their own experiences and values [22]. An European Commission survey showed that, nowadays, European citizens reckon that non-human animal welfare is an issue of great importance [23]. Nevertheless, the level of concern varies among European countries, and also differs for animal species and their intended use. For example, people may agree to the use of animals in biomedical research, while being reluctant to use them for developing secondary products, such as cosmetics and furs [24]. In the same way, two out of three (66 %) Europeans considered that experimentation using mice is acceptable if it leads to an improvement in human health and wellbeing [25]. However, a part of society opposes any use of animals based on the statement that treating animals differently simply because they belong to a different species is discrimination (speciesism) [26] and a new framework based on principles of justice and compassion has been proposed [27].

In this study we aimed to understand the perception that people, who working with laboratory rodents in biomedicine in Spain, have on a number of issues related to welfare and wellbeing.

## Results

### Participants

A total of 356 individuals answered the survey. According to their professional role with laboratory rodents, participants were divided into three categories; animal caretakers or technicians (55/15 %), welfare officer and/or veterinarians (38/11 %) and researchers (263/74 %). Gender and age of participants are shown in Table 1. Two out of three participants were women and the average age was 38 years (21–69). Participants worked in research institutes (206/58 %), universities (110/31 %), hospitals (25/7 %), pharmaceutical companies (11/3 %) or contract research organizations (4/1 %) from different parts of Spain (Supplementary Table 1). They worked mostly with mice (325/91.2 %), followed by rats (138/38.7 %), while the use of guinea pigs (7/2 %) and hamsters (6/1.7 %) was less common. According to self-reported weekly hours working directly with animals, animal caretakers/technicians worked 30 h ± 11, welfare officers/veterinarians 20 h ± 14 and researcher 10 h ± 10 per week.

**Table 1:** Demographic information of participants

	Gender			Total	Age			
	Male (Cis/Trans)	Female (Cis/Trans)	Prefer not to say	n	21-35	36-49	50-69	Mean ± SD
Animal caretaker or technician	15	40	0	**55**	21	25	9	**39 ± 11**
Welfare officer and/or veterinarian	13	24	1	**38**	3	19	16	**48 ± 9**
Researcher	79	175	9	**263**	144	86	33	**36 ± 10**
**Total**	**107**	**239**	**10**	**356**	**168**	**130**	**58**	**38 ± 11**

Number of subjects according to job category, gender and age

### Environmental enrichment

**Fig. 1:**
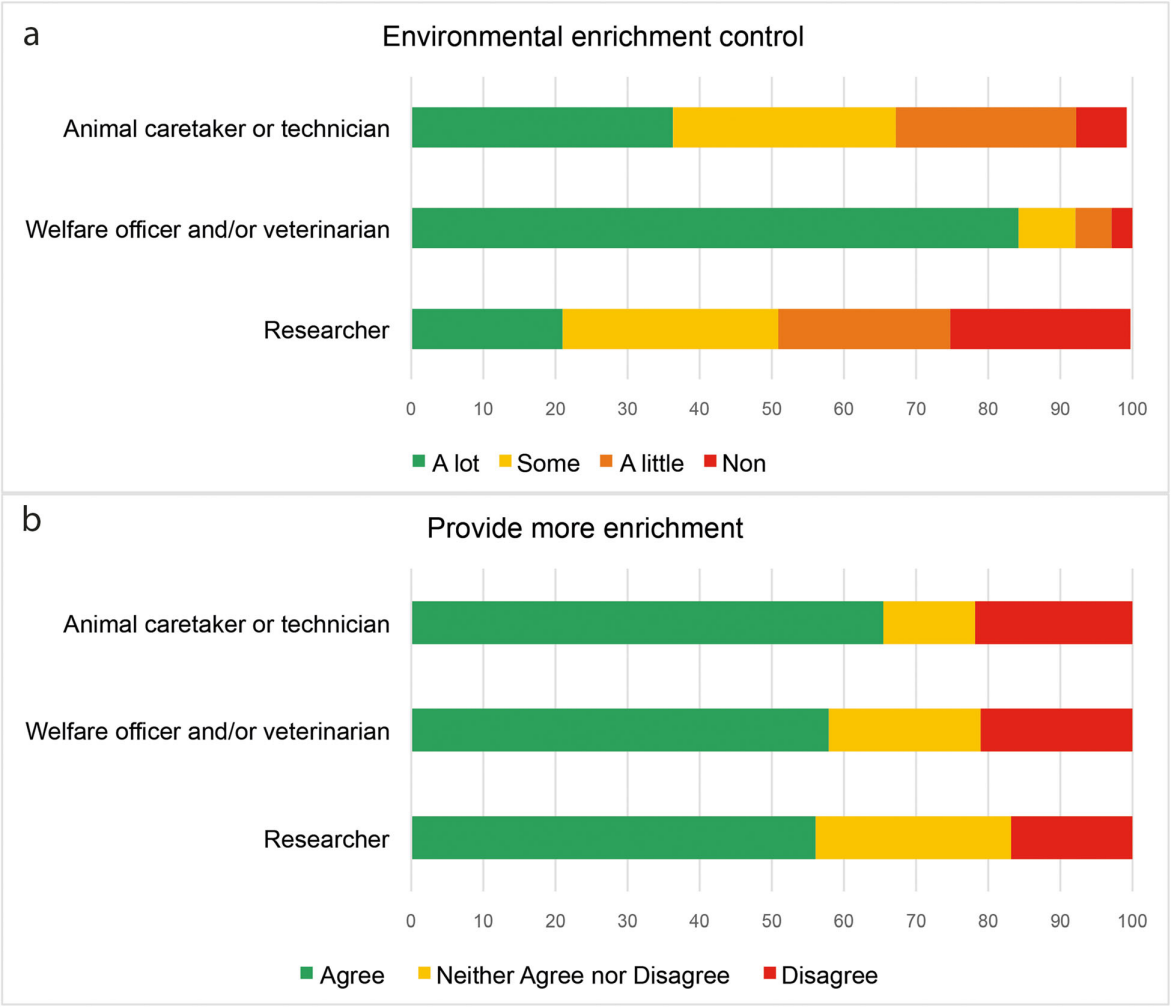
Environmental enrichment. Reported (**a**) degree of control over the type or amount of environmental enrichment provided, and (**b**) the desire to provide more environmental enrichment to their animals, in percentages

All animal facility staff answered affirmatively to the question about the use of environmental enrichment in their animal facilities. Unexpectedly, a few researchers answered that they did not know (26/10 %) or that their animals did not receive enrichment (24/9 %). Regarding control over the type or amount of enrichment provided, some caretakers/technicians (20/36 %) and researchers (55/21 %) reported to have a “lot of control” (Fig. 1a). Nevertheless, 178 participants (60 %) wished they could provide more enrichment (Fig. 1b).

### Animal stress and pain

**Fig. 2:**
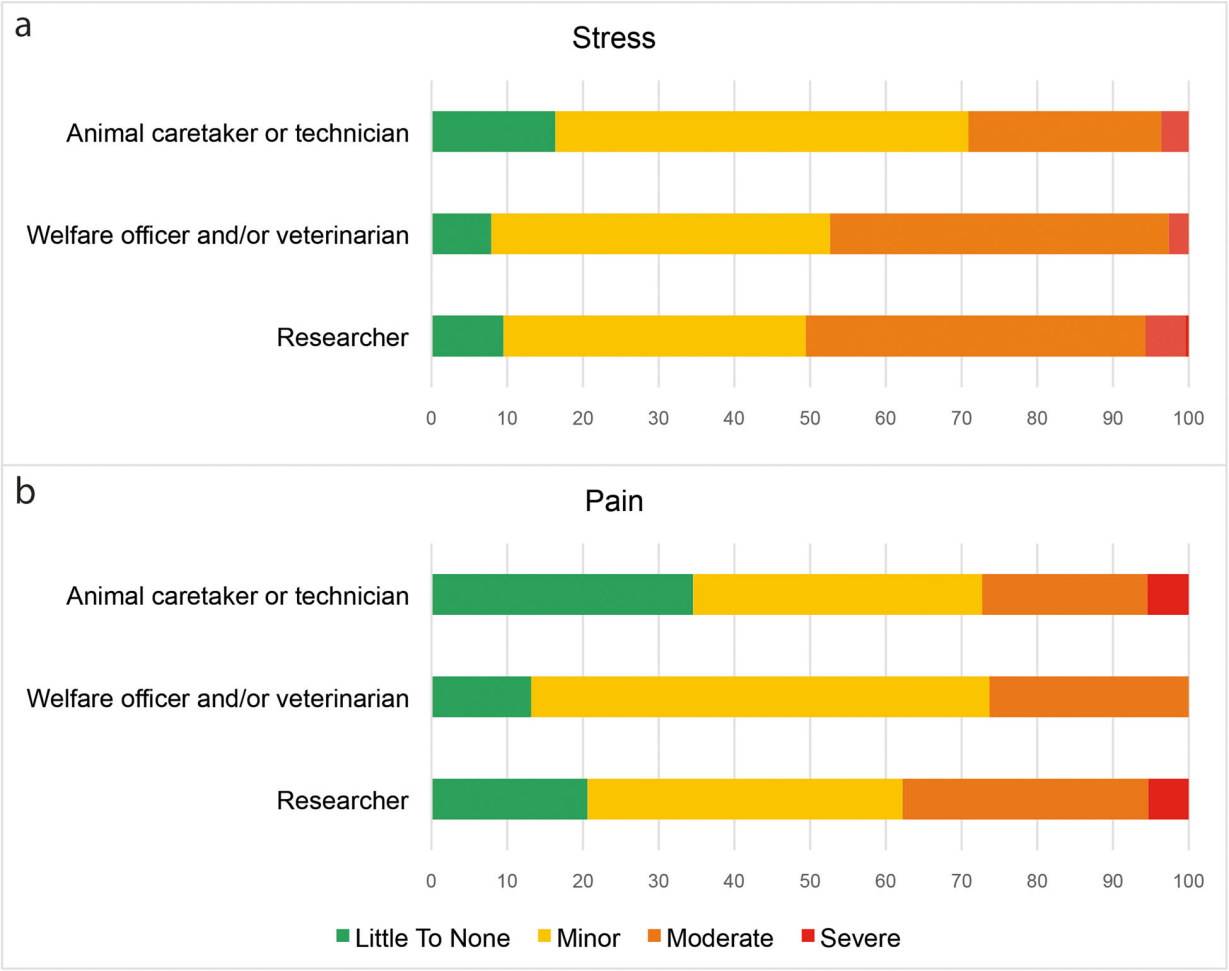
Animal stress and pain. Reported perception about levels of (**a**) stress and (**b**) pain, in percentages

We asked participants to self-asses the degree of stress that their animals suffer and 38 reported “little to none” (10.7 %), 152 “minor” (42.7 %), 149 “moderate” (41.2 %) and 17 “severe” (4.8 %) (Fig. 2a). Youngest participants (21–35 years) reported the highest stress severity (X^2^_(8)_ = 20.213; p = 0.010; Phi = 0.239). Regarding pain, 79 reported “little to none” (22.2 %), 153 “minor” (43 %), 107 “moderate” (30.1 %) and 17 “severe” (4.8 %) (Fig. 2b). A total of 244 participants (68.5 %) reported the use of analgesics, of which 155 commonly used buprenorphine (65.3 %) and 97 meloxicam (39.8 %). Of the 333 participants (93.5 %) that reported the use of anesthetics, 243 used isoflurane (72.9 %), 155 ketamine/xylazine (46.5 %), and 87 ketamine/medetomidine (26.2 %). Overall, the use of local anesthesia was less common (88/24.7 %), being lidocaine (52/60 %) the most used drug in this category (Supplementary Table 2).

### Euthanasia

**Fig. 3:**
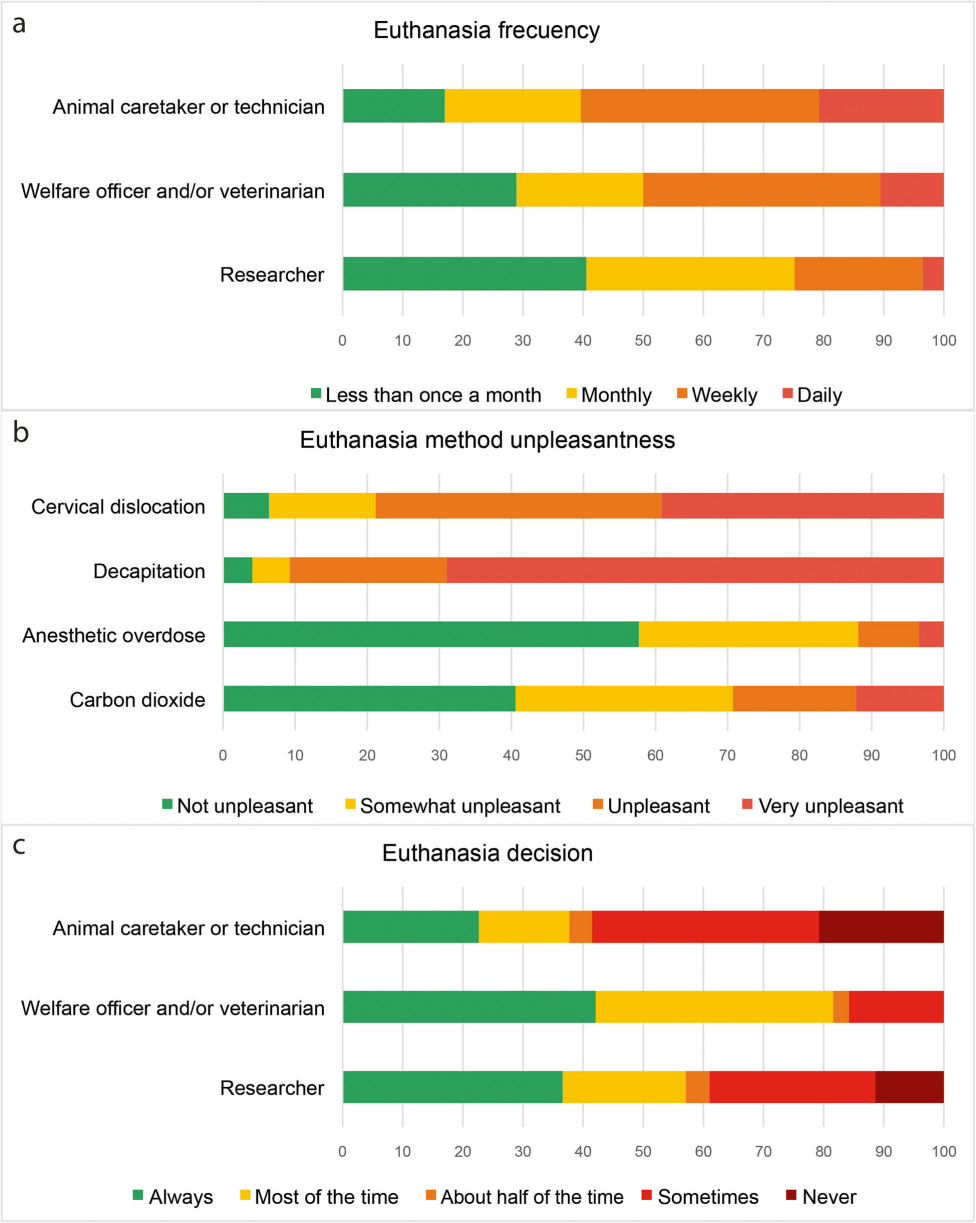
Euthanasia. Reported euthanasia (**a**) frequency, **b** method unpleasantness, and **c** decision, in percentage

A total of 345 participants (96.9 %) had euthanized a rodent. The vast majority of researchers euthanized animals “less than once a month” or “monthly” (191/72.6 %), whereas 19 welfare officers/veterinarians (50 %) and 32 caretakers/technicians (58.2 %) perform it on a “daily” or “weekly” basis (Fig. 3a). Young participants (21–35 years) euthanized rodents less often than the other age categories (X^2^_(2)_ = 8.677; *p* = 0.013; Phi = 0.157). Carbon dioxide (207/60.4 %) was the most used killing method, followed by cervical dislocation (200/58.3 %), anesthetic overdose (126/36.7 %) and decapitation (44/12.8 %). The most unpleasant was decapitation and the least one anesthetic overdose (Fig. 3b). A small percentage of animal caretakers/technicians (11/21 %) and researchers (29/11,4 %) reported that they were never given the choice not to euthanize the rodents they work with (Fig. 3c). The commonest death confirmation method was exsanguination (226/66 %), followed by neck dislocation (193/56.3 %), confirmation of the onset of *rigor mortis* (120/35 %), confirmation of permanent cessation of the circulation (96/28 %) and destruction of the brain (19/5.5 %). Despite being mandatory, 24 participants (7 %) said they did not use any confirmation method.

### Interaction with their animals

**Fig. 4:**
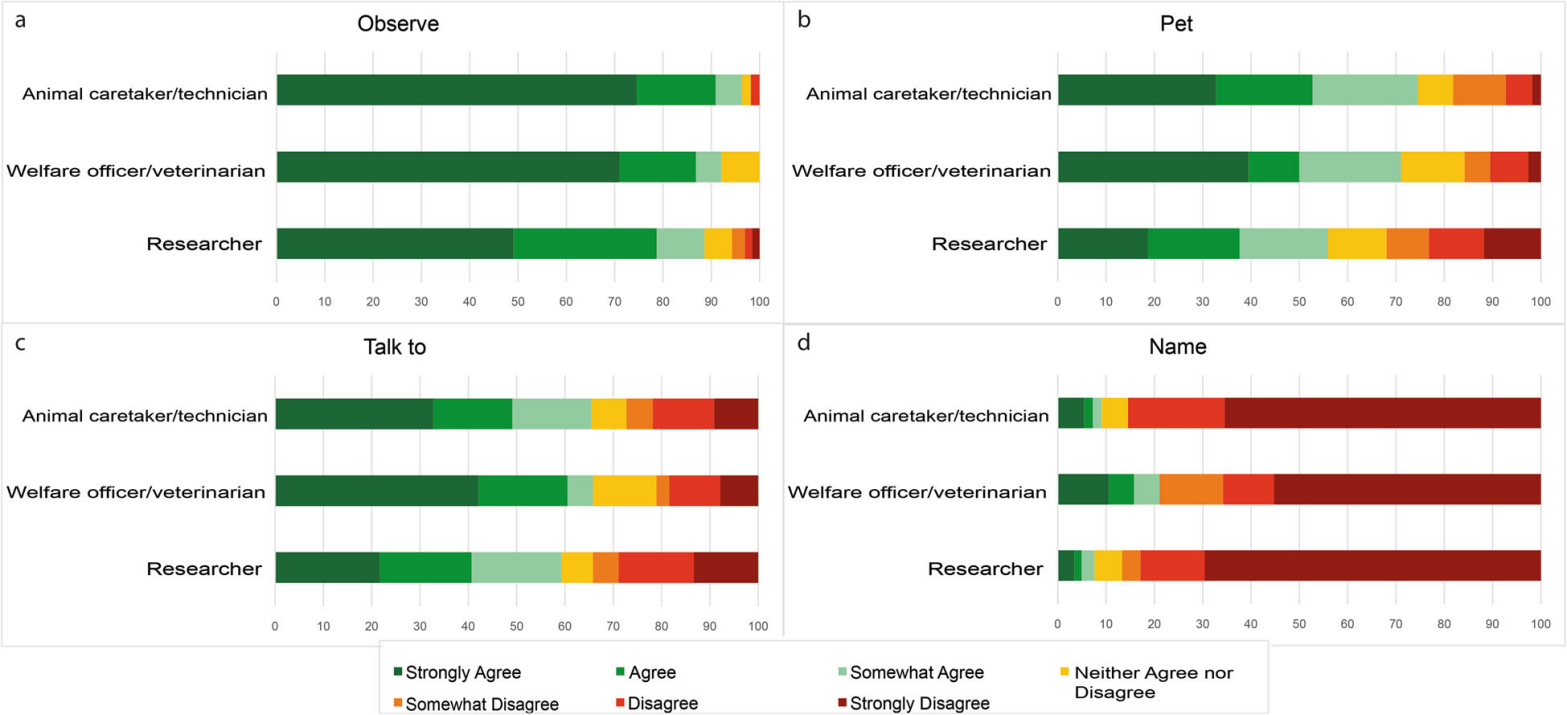
Interaction with their animals. Reported answers about how strongly the participants agreed or disagreed to (**a**) observe, **b** pet, **c** talk to or **d** name their laboratory rodents, in percentages

 Participants were asked about how strongly they agreed or disagreed about how often they observed, pet, talk to or name their laboratory rodents (Fig. 4a-d). Women reported higher interaction with their animals than men (18.6 ± 5 vs. 15.6 ± 4.7; *X*^*2*^_(2)_ = 21.340, *p* < 0.001). The analysis also showed significant differences in the total score by job category (*X*^*2*^_(2)_ = 13.618, *p* = 0.001). Researchers showed lower human-animal interaction than animal caretakers/technicians (16.8 ± 5.1 vs. 18.6 ± 4.5; Mann Whitney *U* = 5628; *p* = 0.010; *r* = 0.22) and welfare officers/veterinarians (16.8 ± 5.1 vs. 19.5 ± 4.9; Mann Whitney *U* = 3511; *p* = 0.003; *r* = 0.30). Our results also indicated a positive low correlation (*rho* = 0.23; *p* < 0.0001) between the total time spent working directly with animals (hours/week) and total human-animal interaction score.

### Social support

**Fig. 5:**
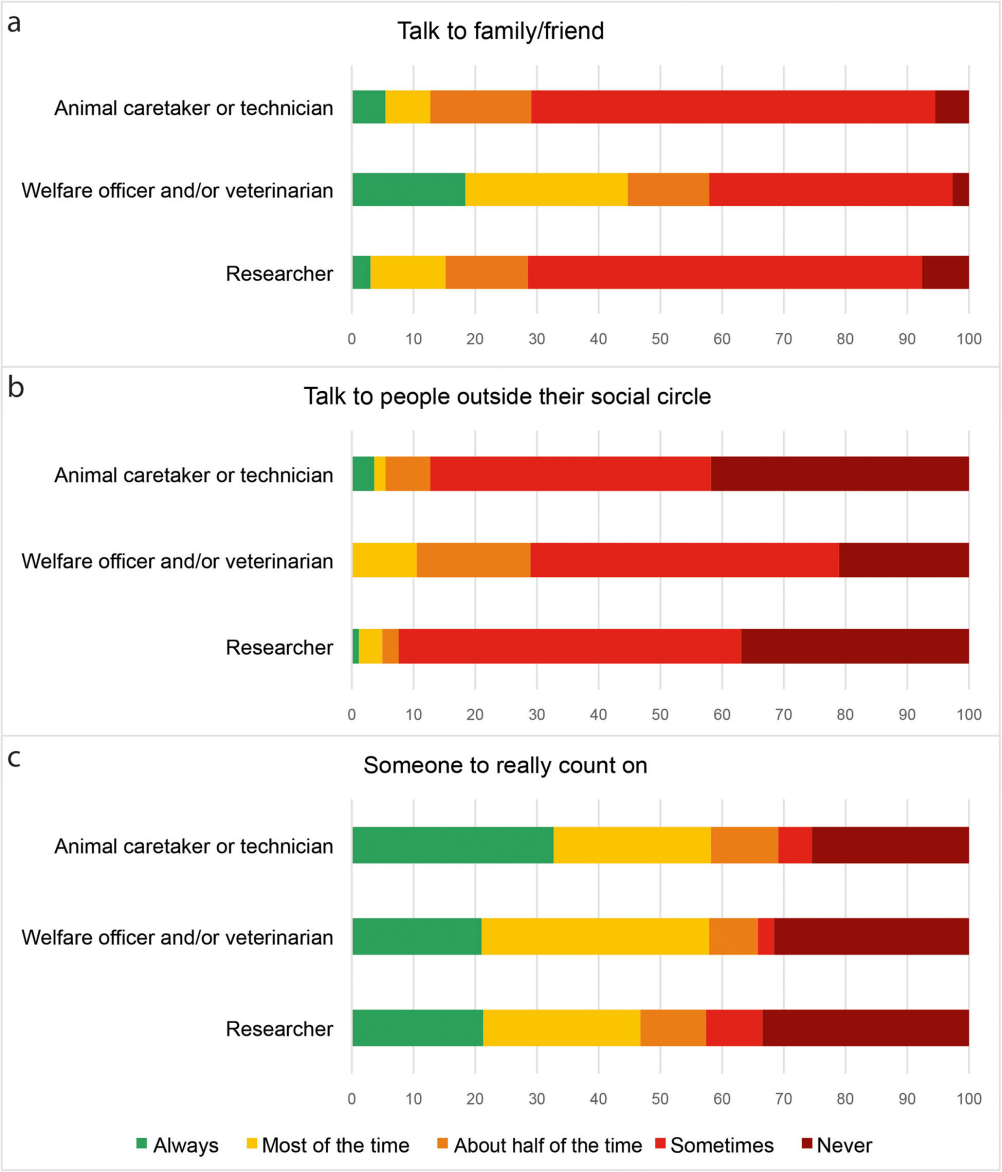
Social support. Reported answers about how often participants talked about laboratory work with rodents to (**a**) their family or friends, (**b**) people outside their social circle and (**c**) how often they felt they had someone to really count on, in percentages

Total social support score showed significant differences according to the job category (*X*^*2*^_(2)_ = 11.245, *p* = 0.004). Welfare officers/veterinarians showed higher scores than animal caretakers/technicians (8.8 ± 2.6 vs. 7.8 ± 2.3; Mann Whitney *U* = 787; *p* = 0.040; *r* = 0.25) and researchers (8.8 ± 2.6 vs. 7.3 ± 2.2; Mann Whitney *U* = 3402; *p* = 0.001; *r* = 0.32). Animal caretakers/technicians and researchers talked to their relatives or friends less often (Fig. 5a). Most participants, 48 animal caretakers/technicians (87.3 %), 27 welfare officers/veterinarians (71.1 %) and 243 researchers (92.4 %) talked “never” or “sometimes” about their work with laboratory rodents to people outside their social circle; (Fig. 5b). Finally, animal caretakers/technicians and welfare officers/veterinarians reported that they feel that they have someone they can really count on when they are dealing with stress at work more often than researchers (Fig. 5c).

## Discussion

Reducing the suffering of animals through refinement techniques is an important component of good scientific practice. This study has allowed us to know the perception about animal welfare, interaction with their animals and social support of laboratory rodent users in Spain.

Participants were divided into three job categories according to their role working with laboratory rodents. Animal caretakers and technicians are in charge of the daily care and husbandry of animals, welfare officers and veterinarians are responsible for enforcing animal welfare legislation in their facilities and researchers use them as a tool in their studies. Participants worked mostly with mice, as expected according to the report on the use of animals in experimentation and other scientific purposes, including teaching in Spain [28].

Animal facilities have standardized their husbandry protocols to reduce research data variability and increase laboratory animals’ welfare. Accordingly, our participants reported high levels of environmental enrichment and low levels of stress. These results indicate that animal care and husbandry programs in Spain are well designed and implemented. However, the responses of the youngest participants and of some researchers could indicate insufficient training or knowledge. As expected, control over the type or amount of enrichment was in welfare officers and/or veterinarians’ hands, but, in some cases, animal caretakers/technicians and researchers participated in the decisions. As reported in a previous study in North America [21], over half of the participants wished they could provide more enrichment to their animals.

Effective management of pain requires knowing the nociceptive pain of the species you work with. For instance, we have found sex and age differences in C57BL6/J mice [29]. Almost half of the participants reported *little to none* or *minor* pain, a result that is in line with the annual report on procedures severity in Spain [28]. Although it is clear that the choice of drug depends on the procedure/surgery, we wanted to know which were the drugs most frequently used by our participants. Buprenorphine continues to be the most widely used analgesic drug followed by meloxicam [10–12], and the most widely used inhalation anesthetic is isoflurane, followed by ketamine in different combinations for parenteral anesthesia [12]. The use of barbiturates was less common than that reported in a previous work [11], indicating a trend to use safer drugs during surgical interventions.

As previously reported, animal facility staff euthanized rodents more frequently by carbon dioxide or cervical dislocation [12, 16, 21, 30]. For euthanasia control, our results are in line with a previous a study which indicates that animal caretakers and technicians have less control but do it more frequently [21]. Decapitation continues to be considered the most unpleasant method and also the least used [12, 16, 21, 30]. Although anesthetic overdose is the least unpleasant method participants reported to perform it less often than cervical dislocation, maybe for economic reasons.

The only difference we observed between genders was human-animal interaction, in line with previous studies (see [31]). By job category, welfare officers and/or veterinarians showed higher total score in human-animal interaction, even if we found a correlation between this score and the time spent with the animals. A previous study showed that animal facilities personnel that reported high levels of interaction with laboratory animals also reported higher levels of compassion satisfaction. This could indicate a greater satisfaction from their close relationship with their animals but it can turn to negative feelings when research procedures cause pain or distress in their animals [21].

Animal facility personnel reported moderate levels of social support, as previously reported [21] and researchers showed the lowest score. The vast majority of participants never talked about their work with laboratory rodents to people outside their social circle. This may indicate that the use of animals in research is considered a sensitive topic in Spain.

## Conclusions

Overall, the participants showed high awareness and sensitivity to rodent wellbeing; animal facility staff reported a similar perception on welfare questions, independently of their category, while researchers, who spent less time with the animals, showed less awareness and manifested lower human-animal interaction and less social support. Regarding the perception on social acceptance of laboratory animal work, all groups were cautious and rarely talked about their job, suggesting that it is considered a sensitive issue in Spain.

## Methods

### Ethical approval

 All procedures and informed consent protocols were approved by the Ethics Committee for Human-related Research (CEISH) of the University of the Basque Country (UPV/EHU); 2020/175 – M10/2020/222.

### Participants and procedure

Participants were recruited by mail trough the email list of the Spanish Society for Laboratory Animal Science (SECAL-L) and direct emails to known laboratory personnel between December 1, 2020 and February 15, 2021. This study was restricted to people working with laboratory rodents in Spain. In a cover letter attached to the questionnaire, participants were informed that the survey data would be used for scientific purposes and that they would remain anonymous. All participants gave their voluntary informed consent prior to completing the short 10-min online questionnaire (Google Drive platform).

### Questionnaire

The survey contained questions related to participant’s gender, age, institution, current professional role, and hours per week working directly with which species of rodent/s.

Participants were asked whether environmental enrichment was provided in their animal facility. If yes, they were asked about their degree of control or influence over the type or amount of enrichment provided (*non*, *a little*, *some* or *a lot*) and if they wished they could provide more enrichment to their animals than they currently did (*disagree*, *neither agree nor disagree* or *agree*).

The questionnaire also contained questions related to analgesia, anesthesia and euthanasia based on our previous work [12]. Participants were asked which analgesic or anesthetic drugs they commonly used. They were also asked whether they had ever euthanized a rodent. If so, they were asked about the frequency, the method (*carbon dioxide*, *anesthetic overdose*, *decapitation* and/or *dislocation*), personal assessment of the unpleasantness of each of these methods (*not unpleasant*, *somewhat unpleasant*, *unpleasant*, *very unpleasant*), if they got to decide whether they have to euthanize the animal they work with (*never*, *sometimes*, *about half of the times*, *most of the times* and *always*), and finally, the death confirmation method/s based on Spanish legislation.

Questions related to personal perception about animal stress and pain, general conduct towards laboratory rodents and social support were translated into Spanish from a published work [21] following a forward-backward design [32]. Each item was translated into Spanish by two bilingual researchers and then the two translations were compared and discussed until a consensus was reached regarding the wording of each item. The back-translation was done by another two bilingual researchers, and again they compared their translations until they reached a consensus. This translation was examined and compared with the original wording to determine whether the items had the same meaning.

Participants were asked to self-assess the degree of stress and pain level for the animals they work with, using categories based off the Spanish legislation (*little to none*, *minor*, *moderate*, *severe* or *unknown*). The interaction of the participants with their animals was assessed by asking them how strongly they agreed or disagreed from 1 (*strongly disagree*) to 7 (*strongly agree*) about how often they observed, pet, talk to or name their laboratory rodents. The maximum score that a participant could obtain was 28. Finally, social support was assessed by questions about support related to their work with laboratory rodents; how often from 1 (*never*) to 5 (*always*) they talked to friends and/or family and to people outside their social circle about their work, and how often did they feel like they had someone they could really count on when dealing with stress related to their work. The maximum score that a participant could obtain was 15.

### Statistical analysis of data

All statistical analyses were performed with Jamovi (1.16.15) and the level of significance was set to *p* < 0.05. Frequency (%) and distribution (mean ± standard deviation) statistics were use to describe the sample. Gender or job category differences related to human-animal interaction or social support were analyzed using Kruskal–Wallis one-way analysis of variance followed by Mann-Whitney U test for post hoc analysis and rank biserial correlation for the effect size. The relation between the hours worked per week and the human-animal interaction was analyzed using bivariate Spearman correlation. Gender and age group effect on ethical issues were analyzed with chi-square and if the results were significant adjusted residuals were observed.

## Supplementary Information

**Additional file 1: Supplementary Table 1.** Percentage of participants’ distribution by Spanish autonomous communities.

**Additional file 2: Supplementary Table 2.** Reported use of analgesic and anesthetic drugs.

## Data Availability

Data of the study will we available upon reasonable request to the PI.
