# Supplementary material for: Working with laboratory rodents in Spain: a survey on welfare and wellbeing
Source: Lab Anim Res. 2021 Jul 27;37:18. doi: 10.1186/s42826-021-00098-w (PMC8314439; doi:10.1186/s42826-021-00098-w)
Supplement: Supplementary file 1 — Additional file 1: Supplementary Table 1. Percentage of participants’ distribution by Spanish autonomous communities. [file 42826_2021_98_MOESM1_ESM.docx]

**Supplementary Table 1. Participants by Spanish autonomous communities**.

|  | Number of participants - Percentage (%) |
| --- | --- |
| Cataluña | 132 - 37.1 |
| Madrid | 65 - 18.3 |
| País Vasco | 58 - 16.3 |
| Andalucía | 32 - 9 |
| Galicia | 22 - 6.2 |
| Comunidad Valenciana | 22 - 6.2 |
| Castilla La Mancha | 11 - 3.1 |
| Navarra | 4 - 1.1 |
| Asturias | 2 - 0.6 |
| Aragón | 2 - 0.6 |
| Castilla León | 2 - 0.6 |
| Murcia | 2 - 0.6 |
| Islas Baleares | 1 - 0.3 |
| Islas Canarias | 1 - 0.3 |
