# Supplementary material for: Working with laboratory rodents in Spain: a survey on welfare and wellbeing
Source: Lab Anim Res. 2021 Jul 27;37:18. doi: 10.1186/s42826-021-00098-w (PMC8314439; doi:10.1186/s42826-021-00098-w)
Supplement: Supplementary file 2 — Additional file 2: Supplementary Table 2. Reported use of analgesic and anesthetic drugs. [file 42826_2021_98_MOESM2_ESM.docx]

**Supplementary Table 2. Reported analgesic and anesthetic drugs.**

| **Analgesic drug** | |
| --- | --- |
| Buprenorphine | 155 (63.5%) |
| Meloxicam | 97 (39.8%) |
| Acetaminophen | 29 (11.9%) |
| Fentanyl | 27 (11.1%) |
| Carprofen | 26 (10.7%) |
| Ibuprofen | 17 (7%) |
| Morphine | 16 (6.6%) |
| Ketoprofen | 5 (2%) |
| **General anesthetic drug** | |
| Isoflurane | 243 (72.9%) |
| Ketamine + Xylazine | 155 (46.5%) |
| Ketamine + Medetomidine | 87 (26.2%) |
| Sevoflurane | 48 (14.5%) |
| Sodium pentobarbital | 41 (12.3%) |
| Ketamine + Midazolam | 16 (4.9%) |
| Propofol | 16 (4.9%) |
| Fentanyl + Medetomidine | 15 (4.6%) |
| Ketamine + Diazepam | 15 (4.6%) |
| Chloral hydrate | 14 (4.3%) |
| Urethane | 7 (2.2%) |
| Thiopental | 6 (1.8%) |
| **Local anesthetic drug** | |
| Lidocaine | 52 (60%) |
| EMLA-cream | 21 (23.8%) |
| Bupivacaine | 18 (20.8%) |
| Mepivacaine | 3 (3.8%) |
